# mTOR-dependent TFEB activation and TFEB overexpression enhance autophagy-lysosome pathway and ameliorate Alzheimer's disease-like pathology in diabetic encephalopathy

**DOI:** 10.1186/s12964-023-01097-1

**Published:** 2023-05-04

**Authors:** Lizhen Cheng, Yixin Chen, Donghao Guo, Yuan Zhong, Wei Li, Yijia Lin, Ya Miao

**Affiliations:** 1 Department of Geriatrics, Shanghai Sixth People’s Hospital Affiliated to Shanghai Jiao Tong University School of Medicine, 600 Yishan Road, Xuhui, Shanghai, 200233 People’s Republic of China; 2 Division of Cardiology, Department of Medicine and Therapeutics, Faculty of Medicine, The Chinese University of Hong Kong, Shatin, Hong Kong

**Keywords:** Diabetic encephalopathy, Aβ, p-Tau, Neuronal apoptosis, Autophagy-lysosome pathway, TFEB

## Abstract

**Background:**

Diabetic encephalopathy (DE) is a complication of type 2 diabetes mellitus (T2DM) that features Alzheimer's disease (AD)-like pathology, which can be degraded by the autophagy-lysosome pathway (ALP). Since transcription factor EB (TFEB) is a master regulator of ALP, TFEB-mediated ALP activation might have a therapeutic effect on DE, but this has yet to be investigated.

**Methods:**

We established T2DM mouse models and cultured HT22 cells under high-glucose (HG) conditions to confirm the role of ALP in DE. To further investigate this, both mice and HT22 cells were treated with 3-methyladenine (3-MA). We also analyzed the content of TFEB in the nucleus and cytoplasm to evaluate its role in ALP. To confirm the effect of TFEB activation at the post-translational level in DE, we used rapamycin to inhibit the mechanistic target of rapamycin (mTOR). We transduced both mice and cells with TFEB vector to evaluate the therapeutic effect of TFEB overexpression on DE. Conversely, we conducted TFEB knockdown to verify its role in DE in another direction.

**Results:**

We found that T2DM mice experienced compromised cognitive function, while HG-cultured HT22 cells exhibited increased cell apoptosis. Additionally, both T2DM mice and HG-cultured HT22 cells showed impaired ALP and heavier AD-like pathology. This pathology worsened after treatment with 3-MA. We also observed decreased TFEB nuclear translocation in both T2DM mice and HG-cultured HT22 cells. However, inhibiting mTOR with rapamycin or overexpressing TFEB increased TFEB nuclear translocation, enhancing the clearance of ALP-targeted AD-like pathology. This contributed to protection against neuronal apoptosis and alleviation of cognitive impairment. Conversely, TFEB knockdown lessened ALP-targeted AD-like pathology clearance and had a negative impact on DE.

**Conclusion:**

Our findings suggest that impaired ALP is responsible for the aggravation of AD-like pathology in T2DM. We propose that mTOR-dependent TFEB activation and TFEB overexpression are promising therapeutic strategies for DE, as they enhance the clearance of ALP-targeted AD-like pathology and alleviate neuronal apoptosis. Our study provides insight into the underlying mechanisms of DE and offers potential avenues for the development of new treatments for this debilitating complication of T2DM.

**Graphic Abstract:**

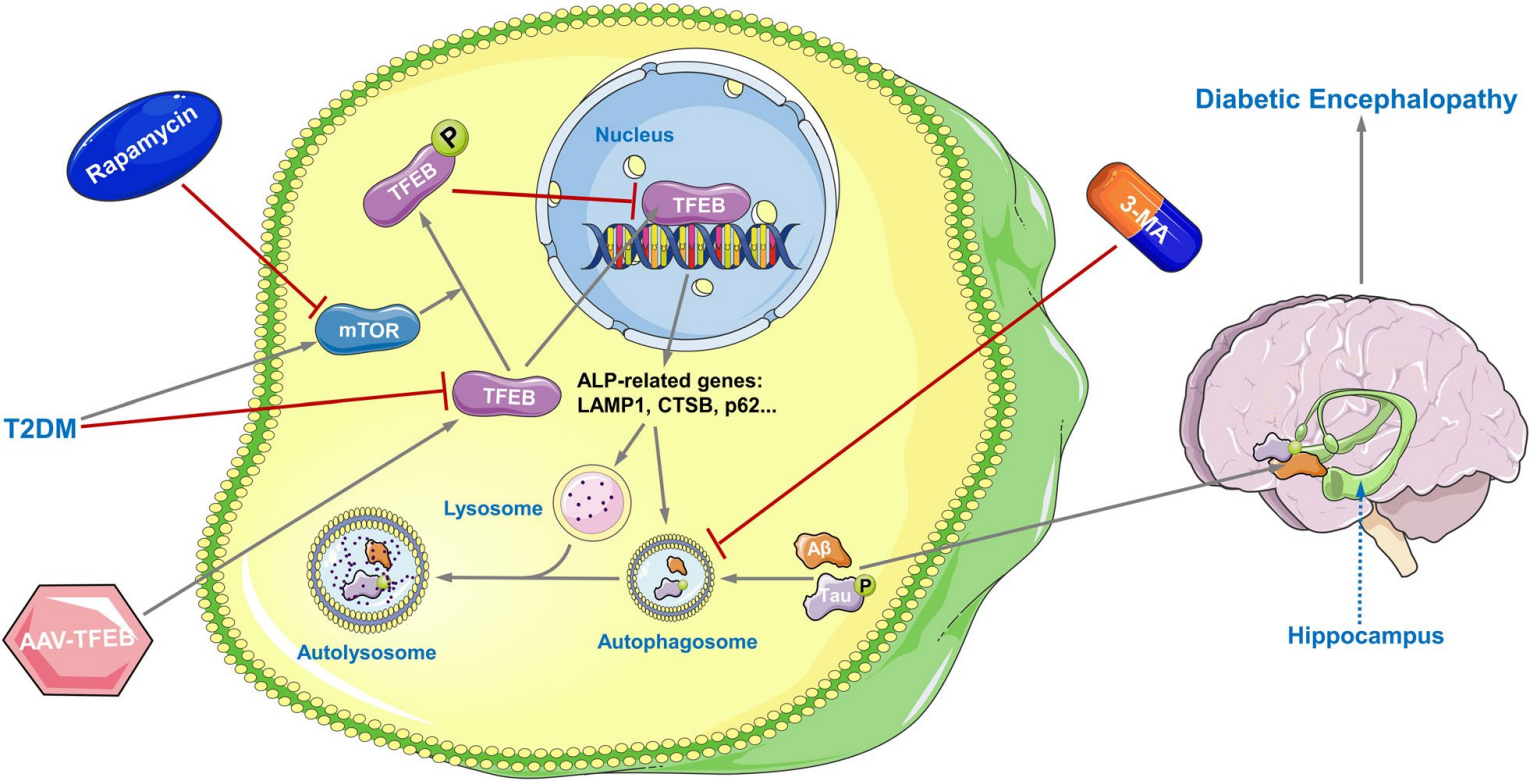

**Video abstract**

**Supplementary Information:**

The online version contains supplementary material available at 10.1186/s12964-023-01097-1.

## Introduction

Diabetic encephalopathy (DE) is a complication of type 2 diabetes mellitus (T2DM) that causes cognitive impairment and structural changes in the brain [1, 2], leading to worsened diabetes management and increased risk of death [3, 4]. Currently, no cure for DE exists, making it crucial to devote significant efforts to research its pathological mechanisms and develop effective therapeutic strategies.

Alzheimer's disease (AD) is characterized by the accumulation of β-amyloid (Aβ) peptides and hyperphosphorylated tau (p-Tau) protein in the form of senile plaques and neurofibrillary tangles, respectively, which cause neurotoxicity and neuronal loss leading to memory and learning impairments [5–7]. Interestingly, the post-mortem brains of patients with T2DM and experimental T2DM models also show an accumulation of Aβ and p-Tau [8–12]. Therefore, this AD-like pathology is considered a crucial pathogenesis of DE, and therapeutic strategies for DE are being developed to target the alleviation of AD-like pathology [13–15].

The autophagy-lysosomal pathway (ALP) is a cellular process that enables cells to degrade intracellular long-lived proteins and organelles via lysosome. ALP deregulation has been linked to various human diseases [16–19]. In the pathophysiology of AD, the importance of ALP in Aβ and p-Tau clearance has been recognized [20–26]. Moreover, impaired ALP has been reported in some regions of the brain, such as the hippocampus and hypothalamus, under diabetic conditions [27]. These findings suggest that impaired ALP might be responsible for the accumulation of Aβ and p-Tau in DE and that ALP activation could be a potential treatment for DE.

Transcription factor EB (TFEB), a member of the MiT/TFE family of transcription factors, is a key regulator of ALP. It coordinates the expression of autophagy and lysosomal targeted genes by translocating into the nucleus [28]. The nuclear translocation of TFEB is determined by its transcriptional level and post-translational modifications. Exogenous overexpression experiments have shown that a higher level of TFEB transcription increases its nuclear translocation [29, 30]. Additionally, post-translational modifications, such as phosphorylation, can also regulate TFEB's nuclear translocation. Suppression of the mechanistic target of rapamycin (mTOR) results in dephosphorylation of TFEB and its accumulation in the nucleus, where it promotes the transcription of its target genes [28, 31]. Therefore, activating ALP by regulating the nuclear translocation of TFEB at the post-translational or transcriptional level could be a viable therapeutic option for DE.

The primary objective of our study is to investigate the therapeutic potential of TFEB in treating DE by activating ALP-mediated clearance of AD-like pathology. Specifically, our in vivo and in vitro studies showed that nuclear translocation of TFEB decreased in the hippocampus of T2DM mice and in high glucose (HG) cultured HT22 cells, while mTOR-dependent TFEB activation at the post-translational level, as well as neuronal-targeted TFEB overexpression at the transcriptional level, can promote ALP-targeted clearance of AD-related proteins such as Aβ and p-Tau, which ultimately resulted in the alleviation of DE. Our study provides evidence that TFEB activation may represent a promising therapeutic strategy for the treatment of DE through the clearance of AD-like pathology.

## Materials and methods

### Animals and T2DM model

Male C57BL/6 mice (6–8 weeks old, weighing 18–22 g) were maintained in a specific pathogen-free (SPF) environment with light and temperature control and had ad libitum access to food and water.

Mice were divided into 13 groups randomly, with 10 mice per group. To establish T2DM model, mice were given a high fat diet (HFD, D12492, Research Diets, Inc, USA). After 12 weeks, mice were injected by streptozocin (STZ, 40 mg/kg body weight for 5 consecutive days [32]; S0130, Sigma-Aldrich, USA) dissolved in a 50-mM citric acid buffer and continuously fed HFD, whereas the control (CON) mice were injected with the citric acid buffer and fed with a normal chow diet. The T2DM model was considered successful if random blood glucose levels over 11.1 mmol/L [33]. A schematic illustration of the timeline and the experimental procedures is shown in Additional file 1: Fig. S1A.

Random blood glucose (Accu-Chek Performa, Roche Diagnostics Unc., Indianapolis, IN, USA) and body weight of mice were monitored weekly during the study. After modeling, intraperitoneal glucose tolerance test (IPGTT) was performed. Cardiac puncture blood collection was performed before animal sacrifice and plasma insulin was then measured using a mice insulin ELISA kit (AIS-32100-96T).

All procedures involving animals and their care were approved (approval ID: DWSY2019-082) by the Animal Experimentation Ethics Committee of Shanghai Jiao Tong University Affiliated Sixth People’s Hospital (SYXK 2016-0020) following the guidelines of the Institutional Animal Care and Use Committee (IACUC) of Shanghai Jiao Tong University.

### Adeno-associated virus (AAV) vector packaging in vivo

For TFEB overexpression, the coding regions of TFEB were amplified from mouse cDNA by real-time quantitative reverse transcription polymerase chain reaction (RT-qPCR) or an empty vector were cloned into the AAV-BBB2.0 plasmids (CMV promoter; Hanbio Biotechnology, Shanghai, China). For TFEB knockdown, a selected shRNA sequence for mice TFEB and a nontarget control were inserted into the AAV-BBB2.0 vector (U6 promoter; Hanbio Biotechnology, Shanghai, China). The shRNA sequences used in this study were: TFEB shRNA: Top strand: 5′-AATTCGCCAAGAAGGATCTGGACTTAATTCAAGAGATTAAGTCCAGATCCTTCTTGGTTTTTTG-3′; Bottom stand: 5′-GATCCAAAAAACCAAGAAGGATCTGGACTTAATCTCTTGAATTAAGTCCAGATCCTTCTTGGCG-3′. Scramble RNA: Top strand:5′-GATCCGTTCTCCGAACGTGTCACGTAATTCAAGAGATTACGTGACACGTTCGGAGAATTTTTTC-3′; Bottom stand: 5′-AATTGAAAAAATTCTCCGAACGTGTCACGTAATCTCTTGAATTACGTGACACGTTCGGAGAACG-3′. The vectors were confirmed by sequencing.

The packaged AAVs were at the following titers: AAV-TFEB-overexpression (TFEB, 1.5 × 10^12^), AAV-NULL (NULL, 1.6 × 10^12^), TFEB shRNA (shTFEB, 1.6 × 10^12^) and AAV-nontarget shRNA (shNT, 1.7 × 10^12^) genome copies per milliliter. The AAV-BBB2.0 vector was delivered to the brain via tail vein injection (100 μL each), as this specific AAV type can cross the brain blood barrier [34, 35].

### Animal treatment

All interventions were performed the time when successfully modeling. To confirm the association between ALP and AD-like pathology, T2DM mice were treated with 3-methyladenine (3-MA, intraperitoneal injection, 30 mg/kg, 3 times/week for 2 weeks [36]; M9281, Sigma-Aldrich, USA). To determine the therapeutic effect of mTOR-dependent TFEB activation, T2DM mice were treated with rapamycin (Rap, intraperitoneal injection, 1 mg/kg/day for 4 weeks [37]; S1039, Selleck, USA). To investigate the therapeutic effect of TFEB overexpression, mice were treated with AAV-TFEB. Besides, AAV-TFEB together with 3-MA treatment was applied to confirm TFEB-mediated ALP activation. Finally, AAV-shTFEB treatment was conducted to further validate the role of TFEB in DE.

After behavioral experiment, the mice were sacrificed. The brain samples were cut into two parts, one of which were further operated for acquiring hippocampus tissue and stored at -80° C for subsequent analysis, the other one of which was fixed in 4% paraformaldehyde (PFA) for 48 h and embedded in paraffin.

### Mice cognitive function test

After 10 weeks of STZ injection, mice cognitive function was assessed using the Morris Water Maze (MWM) test. The platform was firstly placed 1.0 cm above the water surface and the visual platform experiment was performed to test the visual acuity and motor ability of the mice on the first day. Then, platform was placed under 1.0 cm of water and was kept constant in the hidden platform test. During 4 days of hidden platform test, the mice underwent 4 trials a day, alternating among 4 pseudorandom starting points. If a mouse failed to find the platform within 60 s, it was guided to the platform by the researcher and kept there for 15 s. The inter-trial interval was 60 s. The escape latency time was used as a measure in hidden platform test. Probe trial was conducted 24 h after the hidden platform test. During the probe trial, the platform was removed and mice were free to swim in the tank for 60 s. The latency of first arrival to targeted platform, time spent in the targeted platform quadrant and the number of crossing targeted platform were recorded as a measure of probe trial. All the trials were recorded by a video camera mounted on the ceiling, and data were analyzed using the SMART V3.0 tracking system.

### Cell culture and hyperglycemic stimulation

Mouse hippocampal neuron cells HT22 (Beijing Chuanglian Biotech Institute, Beijing, China) were cultured in Dulbecco's Modified Eagle's medium (DMEM, Life Technologies) with supplemented with 10% fetal bovine serum (FBS, Life Technologies) and incubated at 37 °C with 5% CO_2_.

Optimal growth and survival rate of HT22 cells require 25 mM glucose [38–40]. Hence, the DMEM medium (containing 25 mM basal glucose) cultured HT22 cells was regarded as normal glucose (NG) group. An additional 5 mM, 25 mM or 50 mM glucose was given to evaluate the effect of different glucose concentrations on HT22 cells. In the following experiments, an additional 50 mM was given in HG group [41]. A mannitol (50 mM of D-mannitol) was used as the osmotic control for HG.

### Plasmid constructs and transfection in vitro

Mouse TFEB cDNAs were cloned into the pcDNA3.1 vector (Thermo Fisher, USA) and shTFEB sequence as previous described were cloned into the pLKO.1. HT22 cells were transfected with the aforementioned plasmids or the control plasmids using Lipofectamine 2000 reagent (Invitrogen) according to the manufacturer’s protocol.

### Cell treatment

HT22 cells were cultured either in NG or HG medium in the presence or absence of Rap (5 nM) and 3-MA (3 mM) for 72 h. For TFEB overexpression and knockdown, the corresponding overexpression or interference plasmids were transfected for 24 h. Then, cells treated with different glucose concentrations and the corresponding drugs for 48 h.

### Cell apoptosis assay

Cell apoptosis was determined using an Annexin V/PI Apoptosis Detection kit following the manufacturer’s instructions (40302ES20, Yeasen, China). Cells were incubated with Annexin V-FITC and/or propidium iodide for 30 min in the dark. Then, apoptotic cells were analyzed via flow cytometry (FC500, Beckman Coulter, USA).

### CTSB activity assay

Mice hippocampus tissue samples and HT22 cells were homogenized in a lysis buffer. The homogenates were centrifuged at 12,000×*g* for 10 min at 4 °C, and the supernatant was collected for the measurement of CTSB activity. The protein concentration of the tissue lysates was determined by a BCA Protein Assay Kit (P0012; Beyotime Biotechnology, China). CTSB activity was determined using a fluorometric CTSB activity assay kit (CBA001; Millipore, USA) as described by the manufacturer. The activity of CTSB was expressed as fluorescent units/uM protein.

### TFEB content analysis in nucleus and cytoplasm

Nuclear and cytoplasmic lysates were obtained using NE-PER Nuclear and Cytoplasmic Extraction Reagents according to manufacturer’s instructions (78833, Thermo Fisher Scientific, USA). Western blotting was then performed as described previously. The relative fold differences in expression levels were normalized to the GAPDH or Histone-H3 levels. Densitometry analysis was performed using ImageJ (NIH, USA) software.

### Double immunofluorescence staining

HT22 cells and brain tissue were fixed with 4% PFA. Paraffin-embedded brain sections and cells were washed with PBS and permeabilized with 0.3% Triton X-100 in PBS for 20 min followed by blocking. Then they were incubated overnight at 4 °C with primary antibody Aβ or p-Tau in 0.1% BSA-PBS. On the next day, they were washed 3 times in PBS and then incubated for 1 h at room temperature with fluorochrome-coupled secondary antibody. Then, they were washed 3 times in PBS and incubated overnight at 4 °C with primary antibody LAMP1 in 0.1% BSA-PBS and they were washed 3 times in PBS and then incubated for 1 h at room temperature with fluorochrome-coupled secondary antibody. They were then washed 3 times in PBS for 10 min each and incubated with DAPI (D8417, Sigma, USA) for 30 min at room temperature. Finally, we observed the images under a microscope and picture-taking on a confocal microscope (Leica SPE). Image analysis was performed using ImageJ (NIH, USA) software.

### mRFP-GFP-LC3 fluorescence microscopy

A tandem fluorescent-tagged mRFP-GFP-LC3 adenovirus (Shanghai Asia-Vector Biotechnology, China) was used to infect HT22 cells. After incubating with mRFP-GFP-LC3 adenovirus for 24 h, the cells were subjected to corresponding treatment for an additional 48 h. Next, cells were fixed with 4% PFA, followed by DAPI staining visualizing nuclei. Images were taken on a confocal microscope (Leica SPE). Image analysis was performed using ImageJ (NIH, USA) software.

### Western blotting

The hippocampus tissues and the HT22 cells were homogenized and lysed with a lysis buffer (RIPA with protease and phosphatase inhibitor). Total protein concentrations were determined using a BCA Protein Assay Kit (P0012; Beyotime Biotechnology, China). To prepare cell and animal brain homogenates, protein estimation was performed to ensure equalization, followed by running the samples in the required percentage of gel for the target protein. The transferred target protein was then blotted onto a polyvinylidene difluoride (PVDF) membrane using a BIORAD transfer apparatus. After blocking the membrane in 5% milk for an hour, the membrane was washed twice with Tris-buffered saline with Tween (TBST) solution. The membrane was then incubated with the target primary antibody overnight at 4 °C with constant shaking, after which the primary antibody was collected, and the membrane was washed with TBST. The membrane was subsequently incubated with the target secondary antibody for an hour and washed twice with TBST for 10 min each time. Finally, the bands were visualized by enhanced chemiluminescence method using an electrochemiluminescent solution (Millipore, USA) and the ImageJ (NIH, USA) analysis system. The details of primary antibodies: mTOR rabbit mAb (1:1000; CST-2983; Cell Signaling Technology, USA), p-mTOR rabbit mAb (1:1000; CST-5536; Cell Signaling Technology, USA), TFEB rabbit mAb (1:50; MBS2538354, MyBioSource, USA), p-TFEB (Serin 142) rabbit mAb (1:1000; ABE1971-I; Millipore, USA), LC3 rabbit mAb (1:100; ab192890; Abcam, USA), P62 rabbit mAb (1:1000; ab56416; Abcam, USA), LAMP1 rabbit mAb (1:10,000; ab25630; Abcam, USA), CTSB rabbit mAb (1:1000; ab58802; Abcam, USA), CTSD rabbit mAb (1:1000; ab75852; Abcam, USA), APP rabbit mAb (1:10,000; ab32136; Abcam, USA), Aβ rabbit mAb (1:1000; ab201061; Abcam, USA), Tau rabbit mAb (1:1000; SAB4501830; Sigma-Aldrich, USA), p-Tau rabbit mAb (1:1000; SAB4504557; Sigma-Aldrich, USA), Histone-H3 rabbit mAb (1:1000, ab5103; Abcam, USA) and GAPDH rabbit mAb (1:1000; CST-5174S; Cell Signaling Technology, USA).

### Statistical analyses

Data are presented as the mean ± standard deviation of at least three independent experiments. Student’s t-tests were used to compare two groups, and one-way analysis of variance (ANOVA) followed by Fisher's LSD was used for comparisons of multiple groups. All statistical analyses were performed using GraphPad prism version 8.0.1. Statistical significance was set at *p* < 0.05.

## Results

### The cognition of mice with T2DM was impaired

In the T2DM group, glucose intolerance was observed as indicated by the IPGTT results (Additional file 1: Fig. S1B). Additionally, plasma insulin and random blood glucose levels were significantly increased compared to the control group (Additional file 1: Fig. S1C, D), indicating successful establishment of the T2DM model with insulin resistance, hyperinsulinemia and elevated blood glucose.

**Fig. 1:**
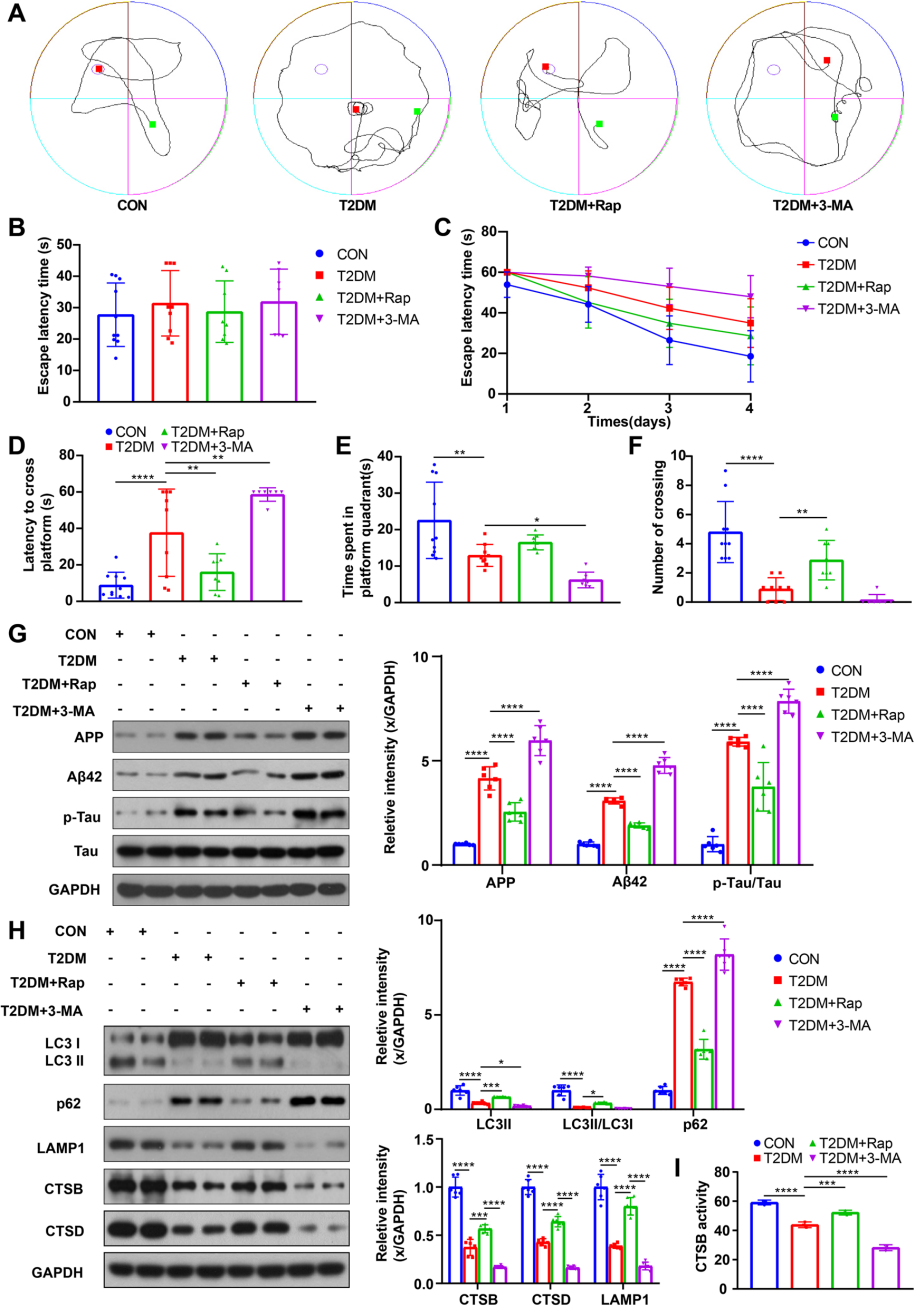
T2DM resulted in cognition impairment and aggravated AD-like pathology in mice, which was related to the dynamic change of ALP function. Typical swimming traces of training days during the hidden platform test (**A**), the starting point was marked by the green square while the ending point was marked by the red square; the escape latency time of visual platform experiment, which showed that there was no obvious movement and visual disturbance in mice of each group (**B**); the escape latency time was analyzed during the hidden platform test (**C**); the latency of first arrival to targeted platform in probe trials (**D**), time spent in the targeted platform quadrant during the probe trials (**E**), the number of crossing targeted platform during the probe trials (**F**) was analyzed; n = 7–10. APP, Aβ42, p-Tau and Tau were measured via western blotting in the hippocampus of mice (**G**); LC3, p62, LAMP1, CTSB and CTSD were measured via western blotting in the hippocampus of mice (**H**); GAPDH was used as the loading control, n = 6. Results of CTSB activity of the mice, the activity of CTSB was expressed as fluorescent units/ug protein (**I**), n = 3. *p < 0.05, **p < 0.01, ***p < 0.001, ****p < 0.0001

Cognitive function was evaluated using the MWM test, which showed no significant differences in escape latency during the visible platform test, indicating no movement or spatial vision disorders in the mice from either group (Fig. 1B). However, during the hidden platform test, the T2DM group had significantly longer escape latency compared to the control group (Fig. 1C). Moreover, the T2DM group showed increased latency of first arrival to the targeted platform and decreased time spent in the targeted platform quadrant and frequency of entrance into the platform zone during the probe trials (Fig. 1D–F), consistent with previous findings [42, 43], indicating impaired spatial memory in T2DM mice.

### Cell apoptosis of HG-cultured HT22 cells was increased

**Fig. 2:**
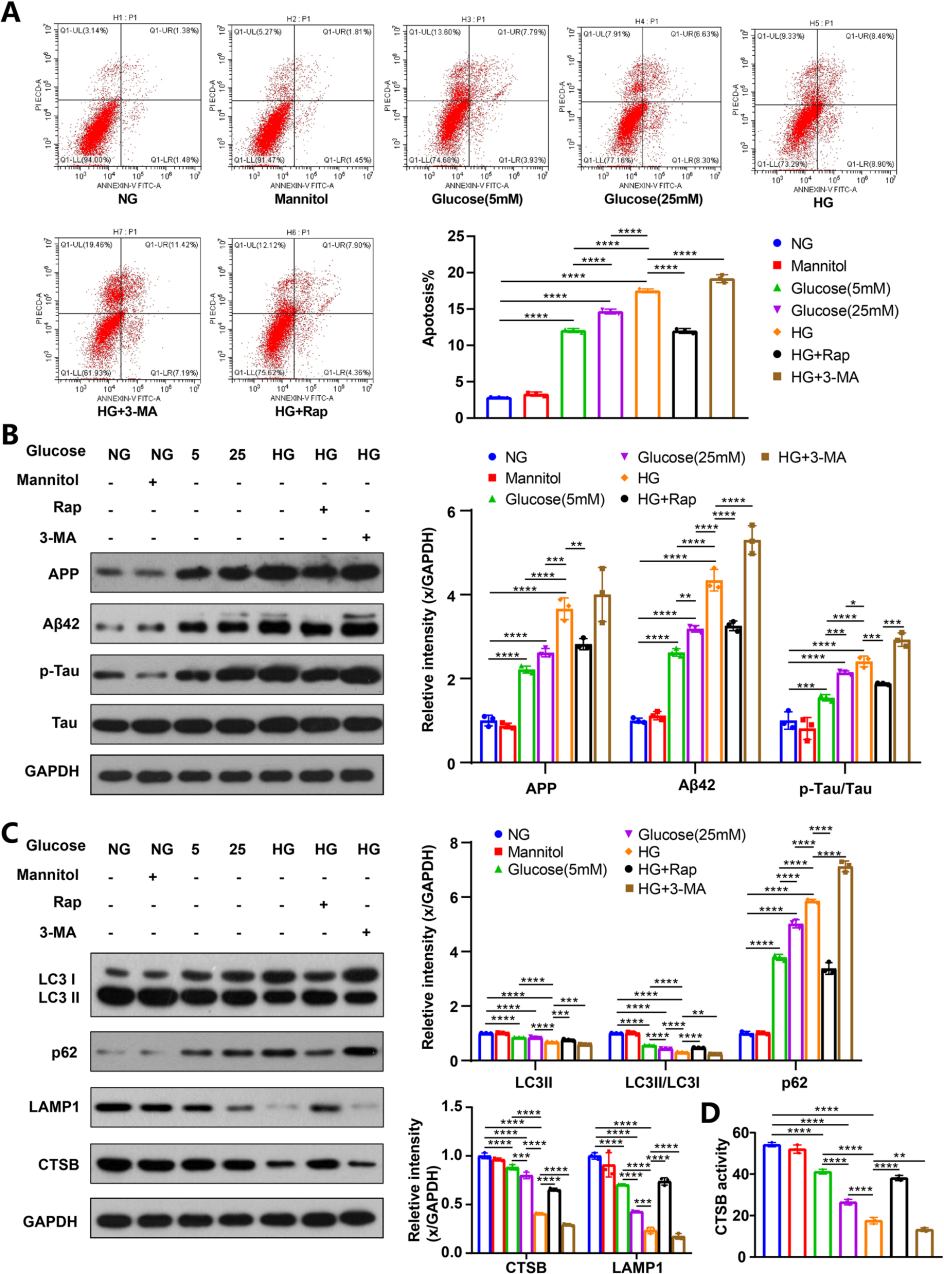
HG increased cell apoptosis and AD-like pathology in HT22 cells, which was related to the dynamic change of ALP function. HT22 cell apoptosis was assayed by flow cytometry, data are presented as the mean ± standard deviation (**A**), n = 3. APP, Aβ42, p-Tau and Tau were measured via western blotting in HT22 cells (**B**); LC3, p62, CTSB and LAMP1 were measured via western blotting in HT22 cells (**C**); GAPDH was used as the loading control, n = 3. Results of CTSB activity of the HT22 cells, the CTSB activity was expressed as fluorescent units/ug protein (**D**), n = 3; *p < 0.05, **p < 0.01, ***p < 0.001, ****p < 0.0001

Stimulation of HT22 cells with various glucose or mannitol concentrations was carried out. The cell apoptosis was elevated in a dose-dependent manner with different glucose concentrations, particularly in the HG group, compared to the NG group (Fig. 2A). Furthermore, there was no significant difference in cell apoptosis between the NG and mannitol groups, thus ruling out the possible role of osmolarity in cell apoptosis.

### Aggravated AD-like pathology and impaired ALP function were observed in T2DM mice and HG-cultured HT22 cells

The deposition of AD-related proteins, such as Aβ and p-Tau, can lead to neuronal apoptosis, which is primarily associated with memory and learning impairment [44, 45]. In our study, we observed a significant increase in the expression of amyloid precursor protein (APP), Aβ42, and p-Tau/Tau ratio in the group with T2DM compared to the control group (Fig. 1G). In vitro experiments further confirmed that the expression of AD-related proteins gradually increased as glucose concentration increased in the culture medium (Fig. 2B).

The ALP is an effective way to degrade AD-related proteins. The levels of microtubule-associated protein 1 light chain 3 (LC3) and sequestosome 1 (p62), which are common autophagy-related markers, were measured in this study. LC3-II and the LC3-II/I ratio are considered to be positively correlated with autophagy function, while the level of p62 is inversely correlated with autophagy activity [46]. In addition, cathepsin B (CTSB), cathepsin D (CTSD) and lysosomal-associated membrane protein 1 (LAMP1) are markers of lysosome mass [47]. Our results showed that the ALP function was significantly inhibited in the hippocampus of mice with T2DM, as demonstrated by an increase in p62 levels and a reduction in LC3II levels, LC3-II/I ratio, CTSB levels, CTSD levels and LAMP1 levels (Fig. 1H). Additionally, CTSB activity was also markedly decreased in T2DM group (Fig. 1I). Furthermore, in vitro experiments further confirmed that high glucose weakened ALP function. As glucose concentration increased, p62 levels increased progressively, while LC3II levels, LC3-II/I ratio, LAMP1 levels, CTSB levels and activity decreased gradually (Fig. 2C, D).

### Impaired ALP was associated with aggravated AD-like pathology

To investigate the role of impaired ALP in exacerbating AD-like pathology in DE, we treated T2DM mice and HG-cultured HT22 cells with 3-MA, a specific autophagy inhibitor that blocks autophagosome formation and nucleation phase [48, 49].

After 3-MA intervention, we observed further suppression of ALP in T2DM mice and HG-cultured HT22 cells, as evidenced by decreased levels of LC3II, LC3II/I ratio, LAMP1, CTSB and CTSD, increased levels of p62, along with decreased CTSB activity (Figs. 1H, I, 2C, D). As expected, 3-MA treatment significantly increased the expression of AD-related proteins (Figs. 1G, 2B). Moreover, T2DM mice treated with 3-MA exhibited worsened cognitive function (Fig. 1C–F), with no significant difference in IPGTT result, glucose level, and plasma insulin (Additional file 1: Fig. S1B–D). Additionally, 3-MA treatment further increased cell apoptosis in HG-cultured HT22 cells (Fig. 2A). Overall, these findings suggest that impaired ALP is associated with aggravated AD-like pathology in DE and that enhancing ALP function could be a promising therapeutic approach for DE.

### Decreased TFEB nuclear translocation was observed in T2DM mice and HG-cultured HT22 cells

**Fig. 3:**
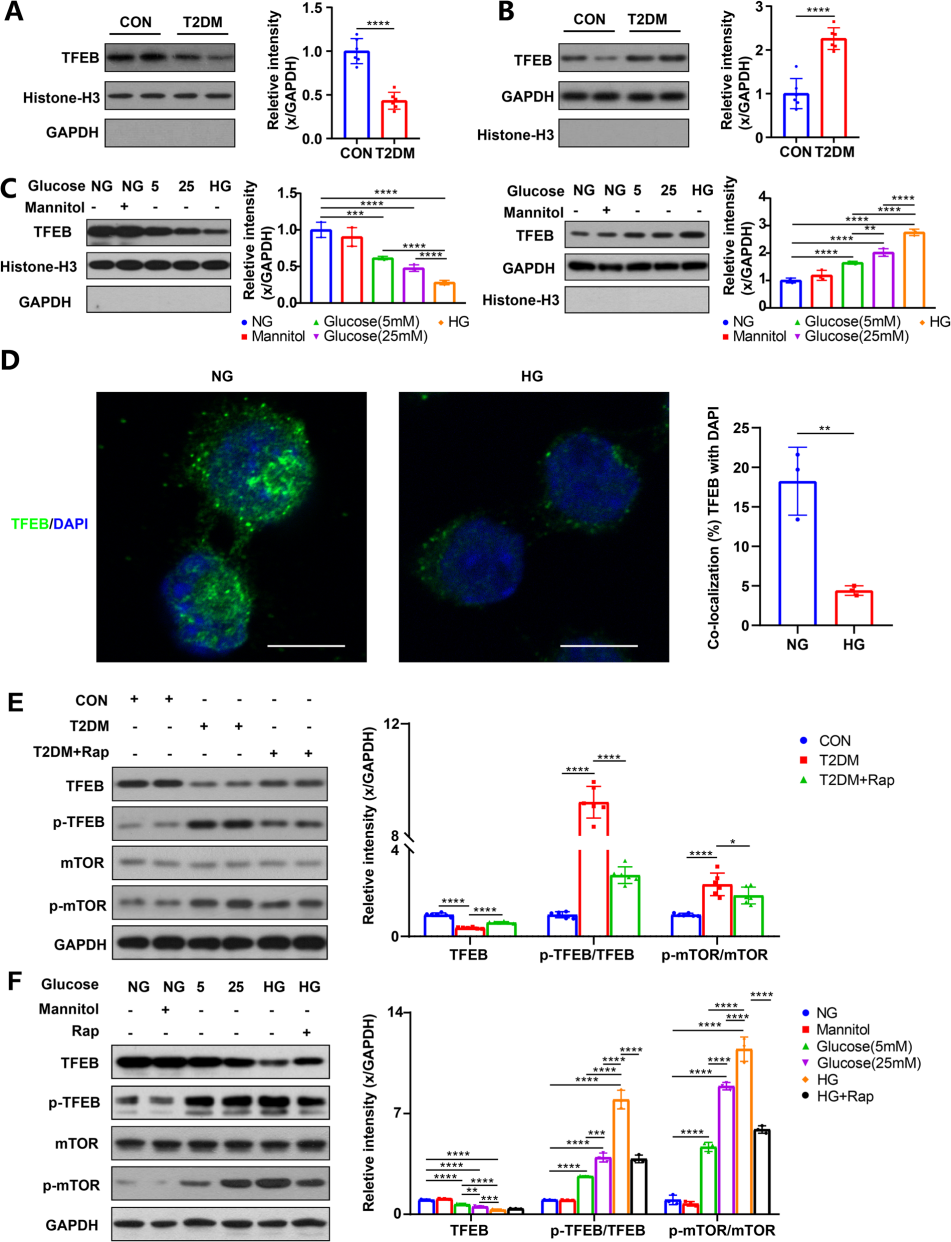
TFEB nuclear translocation was decreased in T2DM mice and HG-cultured HT22 cells, which was related to decreased TFEB activity and expression. Nucleus (**A**) and cytosol TFEB (**B**) were measured via subcellular fractionation experiments by western blotting in the hippocampal of mice, n = 6; Nucleus and cytosol TFEB (**C**) were measured via subcellular fractionation experiments by western blotting in HT22 cells, n = 3; GAPDH was used as the loading control of cytosol TFEB, Histone-H3 was used as the loading control of nucleus TFEB. HT22 cells were treated with or without high glucose. After immunostaining with TFEB (green) and DAPI (blue), cells were examined by fluorescence microscopy, scale bar: 10 μm. The colocalization of TFEB and DAPI was calculated and analyzed, n = 3. TFEB, p-TFEB, mTOR and p-mTOR were measured via western blotting in the hippocampal of mice (**E**), n = 6, and HT22 cells (**F**), n = 3, GAPDH was used as the loading control. *p < 0.05, **p < 0.01, ***p < 0.001, ****p < 0.0001

We hypothesized that TFEB, as a master activator of ALP, is associated with the changes in ALP function in T2DM mice and HG-cultured HT22 cells. Since only TFEB translocation to the nucleus can promote the transcription of ALP-associated genes, we measured TFEB levels in the nucleus and cytoplasm. Compared to the control group, T2DM mice exhibited reduced TFEB levels in the nucleus and increased levels in the cytoplasm (Fig. 3A, B). Similar results were observed in HG-incubated HT22 cells, where increasing glucose concentrations gradually decreased TFEB levels in the nucleus and increased levels in the cytoplasm (Fig. 3C). Immunofluorescence staining further confirm, compared to NG group, TFEB levels in the nucleus were decreased in HG group (Fig. 3D).

Hence, the reduced nuclear translocation of TFEB may contribute to the impaired ALP function in T2DM mice and HG-cultured HT22 cells, and enhancing TFEB nuclear translocation could potentially alleviate DE by activating ALP.

### mTOR inhibition via rapamycin reduced TFEB phosphorylation, activated ALP and attenuated AD-like pathology

Phosphorylation is a post-translational modification of TFEB that determines its nuclear translocation. Only dephosphorylated TFEB can translocate into the nucleus to drive gene expression and lysosome biogenesis. Activated mTOR (p-mTOR) can phosphorylate TFEB, leading to lower TFEB levels in the nucleus [28, 31]. As expected, T2DM mice and HG-cultured HT22 cells showed an increased p-TFEB/TFEB ratio (Fig. 3E, F), and over-activated mTOR was observed in these conditions, as indicated by a higher p-mTOR/mTOR ratio (Fig. 3E, F).

These findings suggest that over-activated mTOR under T2DM or HG conditions contributes to higher phosphorylated TFEB levels, which in turn reduces TFEB levels in the nucleus and inhibits ALP function. Therefore, mTOR inhibition could be a promising strategy for activating ALP and potentially alleviating DE. T2DM mice and HG-cultured HT22 cells were treated with rapamycin, a specific mTOR inhibitor. Rapamycin treatment successfully reduced the p-mTOR/mTOR and p-TFEB/TFEB ratio in vivo and in vitro (Fig. 3E, F), indicating that mTOR activity was inhibited, and phosphorylated TFEB levels were reduced. Furthermore, enhanced ALP function and decreased expression of AD-related proteins were observed in T2DM mice and HG-cultured HT22 cells after rapamycin treatment (Fig. 1G–I; Fig. 2B–D), suggesting that reduced TFEB phosphorylation via mTOR inhibition can activate ALP and attenuate AD-like pathology. Moreover, rapamycin treatment restored cognition in T2DM mice (Fig. 1C–F) without affecting IPGTT results, glucose levels, or plasma insulin (Additional file 1: Fig. S1B–D) and reduced cell apoptosis of HG-cultured HT22 cells (Fig. 2A). These results demonstrate the therapeutic effect of mTOR inhibition on DE by reducing TFEB phosphorylation at the post-translational level.

### TFEB overexpression promoted ALP-targeted clearance of AD-related proteins

In addition to reducing TFEB phosphorylation at the post-translational level, transducing exogenous TFEB is also an effective method to directly enhance TFEB content in the nucleus [29, 30]. Our study found that the total TFEB levels are decreased in T2DM mice and HG-cultured HT22 cells (Fig. 3E, F). Therefore, we conducted both in vitro and in vivo studies to investigate whether TFEB overexpression is an alternative therapeutic avenue for DE.

**Fig. 4:**
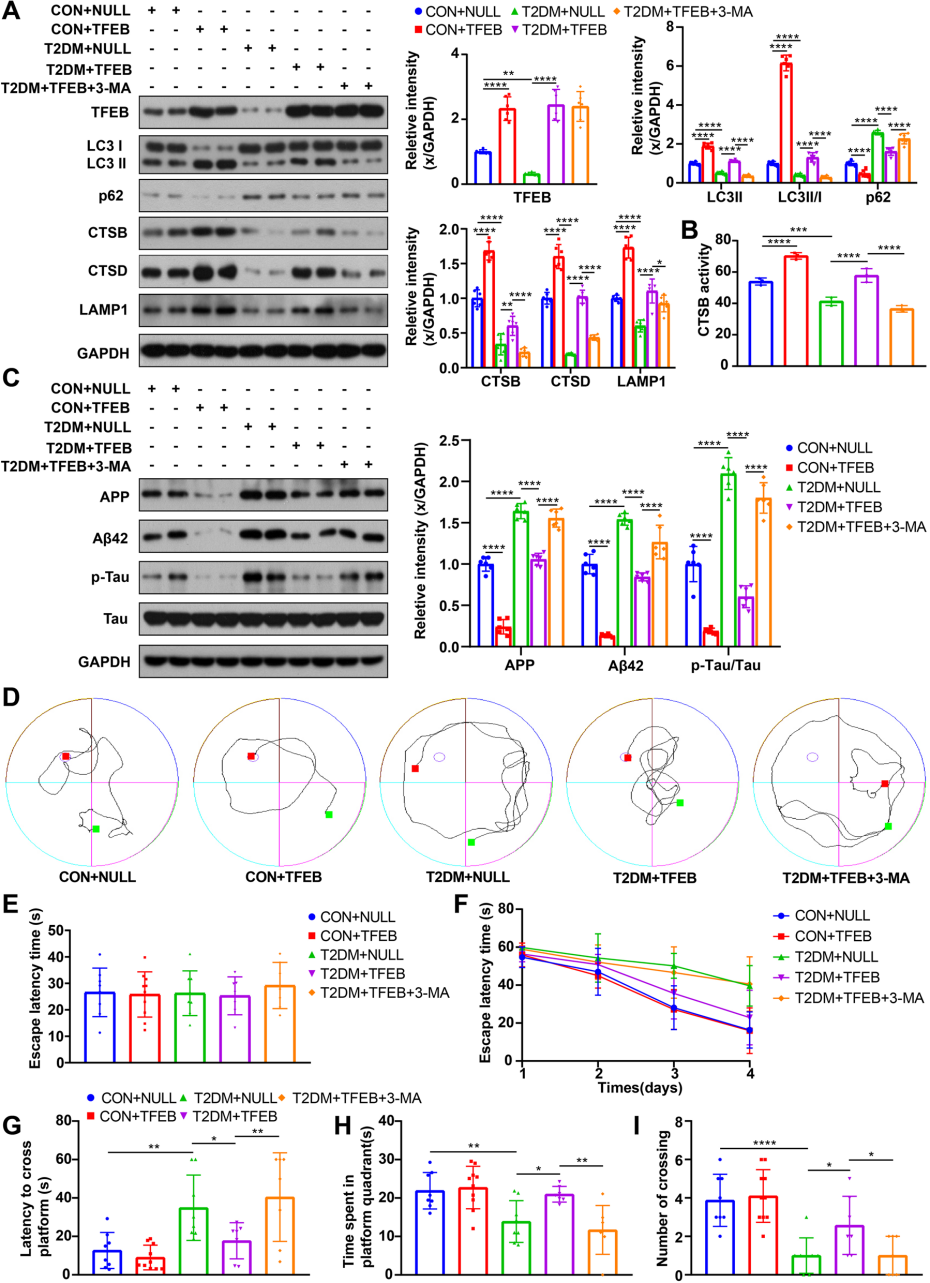
TFEB overexpression enhanced ALP, attenuated AD-like pathology, improved cognitive function of T2DM mice, and the effect of TFEB overexpression was inhibited by 3-MA. TFEB, LC3, p62, CTSB, CTSD and LAMP1 were measured via western blotting in the hippocampus of mice (**A**), GAPDH was used as the loading control, n = 6. Results of CTSB activity of the mice, the activity of CTSB was expressed as fluorescent units/ug protein (**B**), n = 3. APP, Aβ42, p-Tau and Tau were measured via western blotting in the hippocampus of mice (**C**), GAPDH was used as the loading control, n = 6. Typical swimming traces of training days during the hidden platform test (**D**), the starting point was marked by the green square while the ending point was marked by the red square; the escape latency time of visual platform experiment, which showed that there was no obvious movement and visual disturbance in mice of each group (**E**); the escape latency time was analyzed during the hidden platform test (**F**); the latency of first arrival to targeted platform in probe trials (**G**), time spent in the targeted platform quadrant during the probe trials (**H**), the number of crossing targeted platform during the probe trials (**I**) was analyzed; n = 7–10. *p < 0.05, **p < 0.01, ***p < 0.001, ****p < 0.0001

**Fig. 5:**
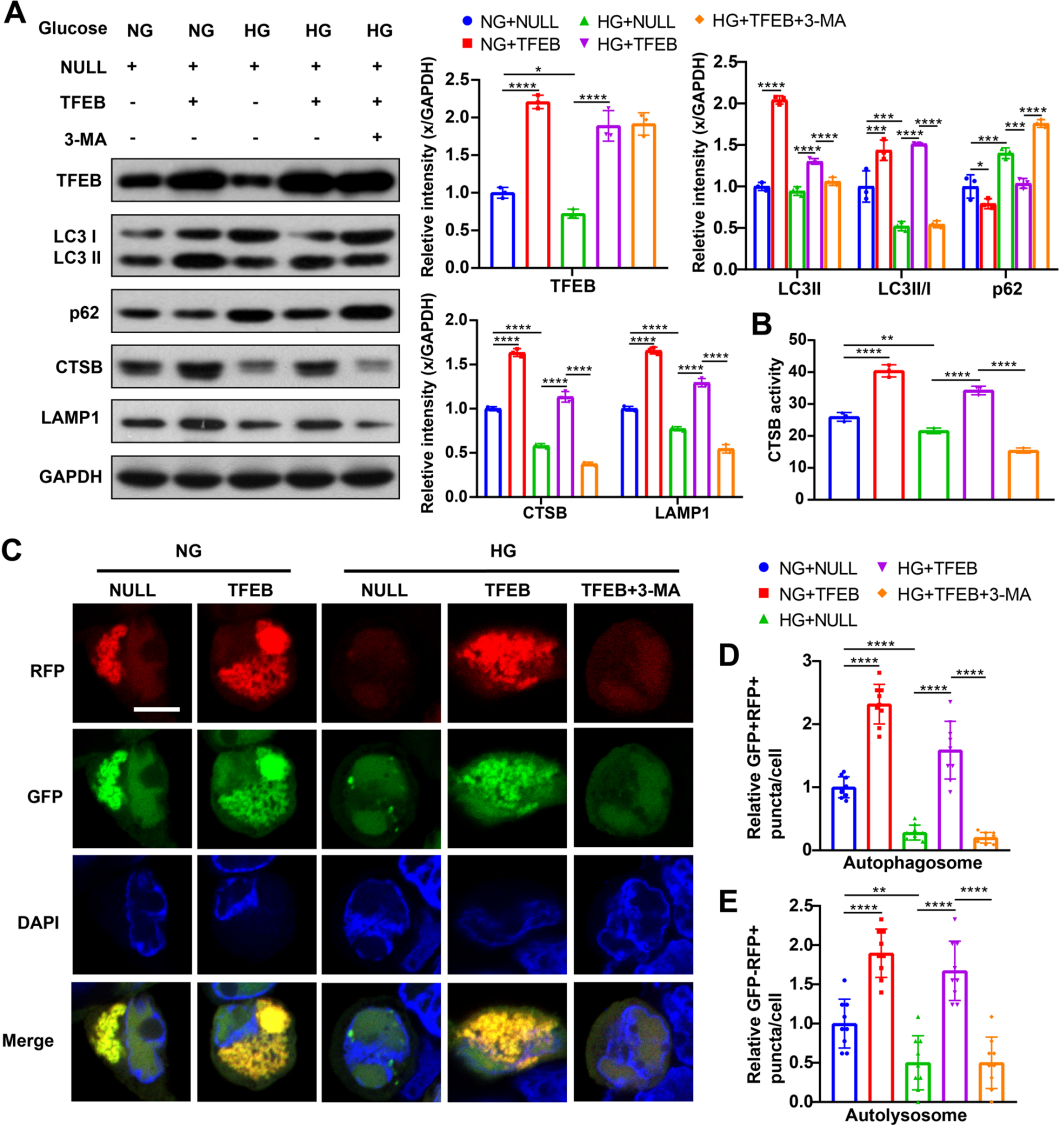
TFEB overexpression enhanced ALP function of HG-cultured HT22 cells and the effect of TFEB was inhibited by 3-MA. TFEB, LC3, p62, CTSB and LAMP1 and were measured via western blotting in HT22 cells (**A**), GAPDH was used as the loading control, n = 3. Results of CTSB activity of the HT22 cells, the activity of CTSB was expressed as fluorescent units/ug protein (**B**), n = 3. Representative confocal images of LC3 puncta in HT22 cells expressing mRFP-GFP-LC3, autophagosomes were labelled by yellow puncta dots due to double staining of green and red fluorescence, autolysosomes were labelled by red puncta only (**C**), scale bar: 10 μm; quantification of relative LC3 puncta was plotted (**D**, **E**) and was normalized to NG + NULL group. n = 9 cells from 3 independent experiments. *p < 0.05, **p < 0.01, ***p < 0.001, ****p < 0.0001

The overexpression of TFEB was confirmed by Western blot analysis in the hippocampus of TFEB-transduced mice (Fig. 4A) and TFEB-transduced HT22 cells (Fig. 5A), which showed a significant increase compared to those transduced with an empty vector. Notably, TFEB overexpression significantly reversed the impaired ALP function in both T2DM mice (Fig. 4A) and HG-cultured HT22 cells (Fig. 5A), as indicated by elevated expression of LC3-II, LC3-II/I ratio, CTSB, CTSD and LAMP1, and a decrease in p62 levels. Consistent with the transcriptional upregulation, CTSB activity also increased after TFEB overexpression in mice (Fig. 4B) and HT22 cells (Fig. 5B).

To further evaluate ALP activation after TFEB overexpression, we used mRFP-GFP tandem fluorescent-tagged LC3 to monitor the synthesis of both autophagosomes and autolysosomes, and to observe changes in the autophagic flux. The GFP signal is quenched in the acidic environment of lysosomes, so autophagosomes are marked with both GFP and RFP, while autolysosomes are only labeled with RFP [50]. Initially, we found that compared to NG-treated cells, the number of autophagosomes and autolysosomes were both decreased in cells challenged with HG, indicating autophagic flux was inhibited at an early stage under HG conditions (Fig. 5C–E). However, the numbers of autophagosomes and autolysosomes were significantly increased after TFEB overexpression in NG or HG-treated HT22 cells, compared to those transduced with an empty vector (Fig. 5C–E). These results suggest that TFEB targeting increased autophagic flux, and that the autophagic flux reduction caused by HG could be recovered by TFEB overexpression.

**Fig. 6:**
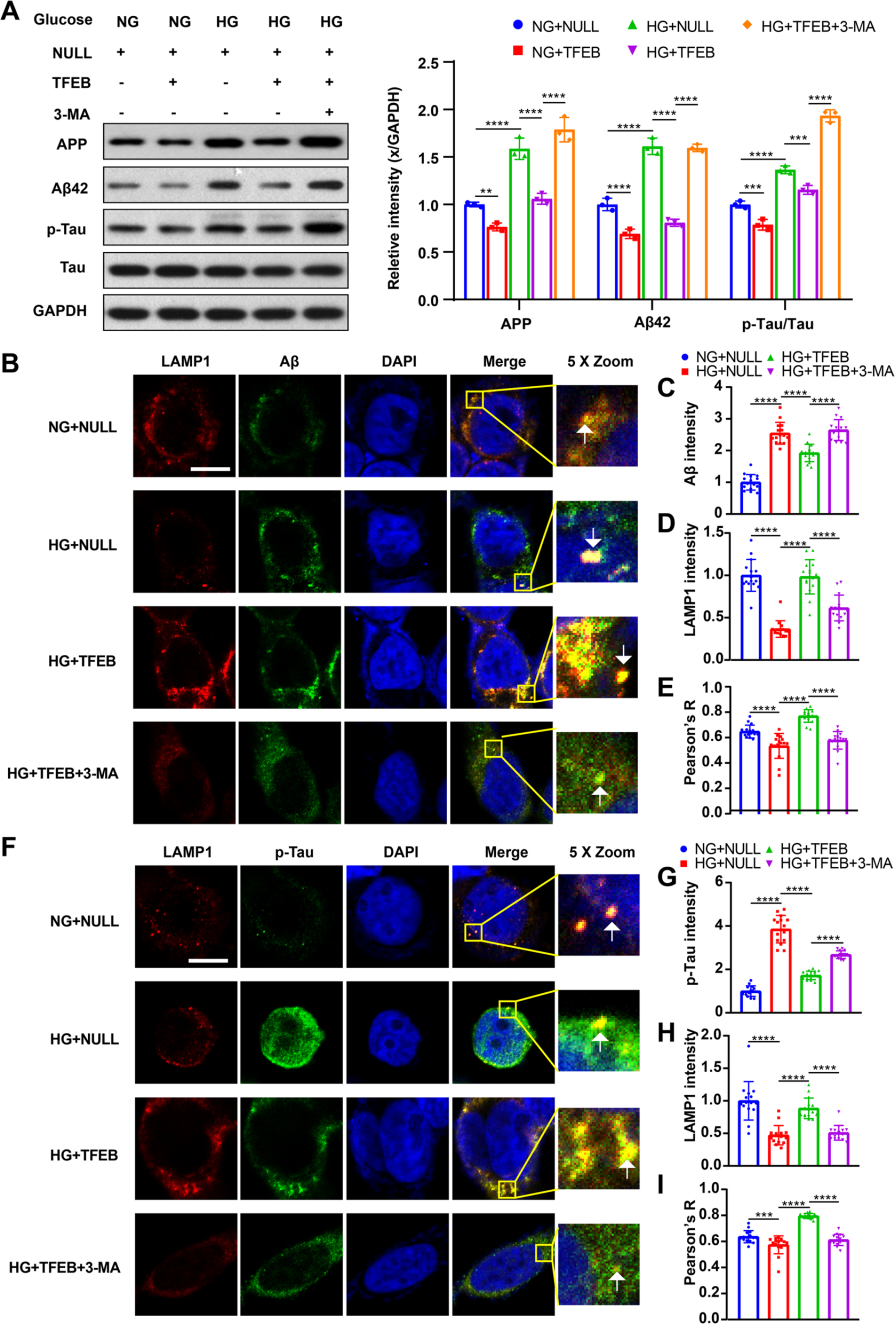
TFEB overexpression promoted clearance of AD-related proteins in HG-cultured HT22 cells and the effect of TFEB overexpression was inhibited by 3-MA. APP, Aβ42, p-Tau and Tau were measured via western blotting in HT22 cells (**A**), GAPDH was used as the loading control, n = 3. Representative double staining image of LAMP1 with Aβ42 in HT22 cells with different treatments (**B**); summary data show LAMP1 and Aβ42 florescent intensities normalized to NG + NULL group (**C**, **D**); the co-localization is represented by yellow signals and quantified as Pearson’s correlation coefficient (**E**). Representative double staining image of LAMP1 with p-Tau in HT22 cells with different treatments (**F**); summary data show LAMP1 and p-Tau florescent intensities normalized to NG + NULL group (**G**, **H**); the co-localization is represented by yellow signals and quantified as Pearson’s correlation coefficient (**I**). scale bar: 10 μm, n = 15 cells from 3 independent experiments. *p < 0.05, **p < 0.01, ***p < 0.001, ****p < 0.0001

We investigated the impact of TFEB overexpression on the AD-like pathology. Our results from western blot analysis and immunofluorescence staining showed a significant decrease in the levels of AD-related proteins such as Aβ42 and p-Tau in mice (Fig. 4C) and cells (Fig. 6A–C, F, G) with TFEB overexpression. Double immunofluorescence staining revealed an increase in the colocalization of LAMP1 with Aβ42 and p-Tau after TFEB overexpression in T2DM mice or HG-cultured HT22 cells compared to those transduced with the empty vector (Additional file 2: Fig. S2A, B, Fig. 6B, E, F, I), indicating successful induction of ALP-targeted clearance of AD-related proteins by TFEB overexpression.

**Fig. 7:**
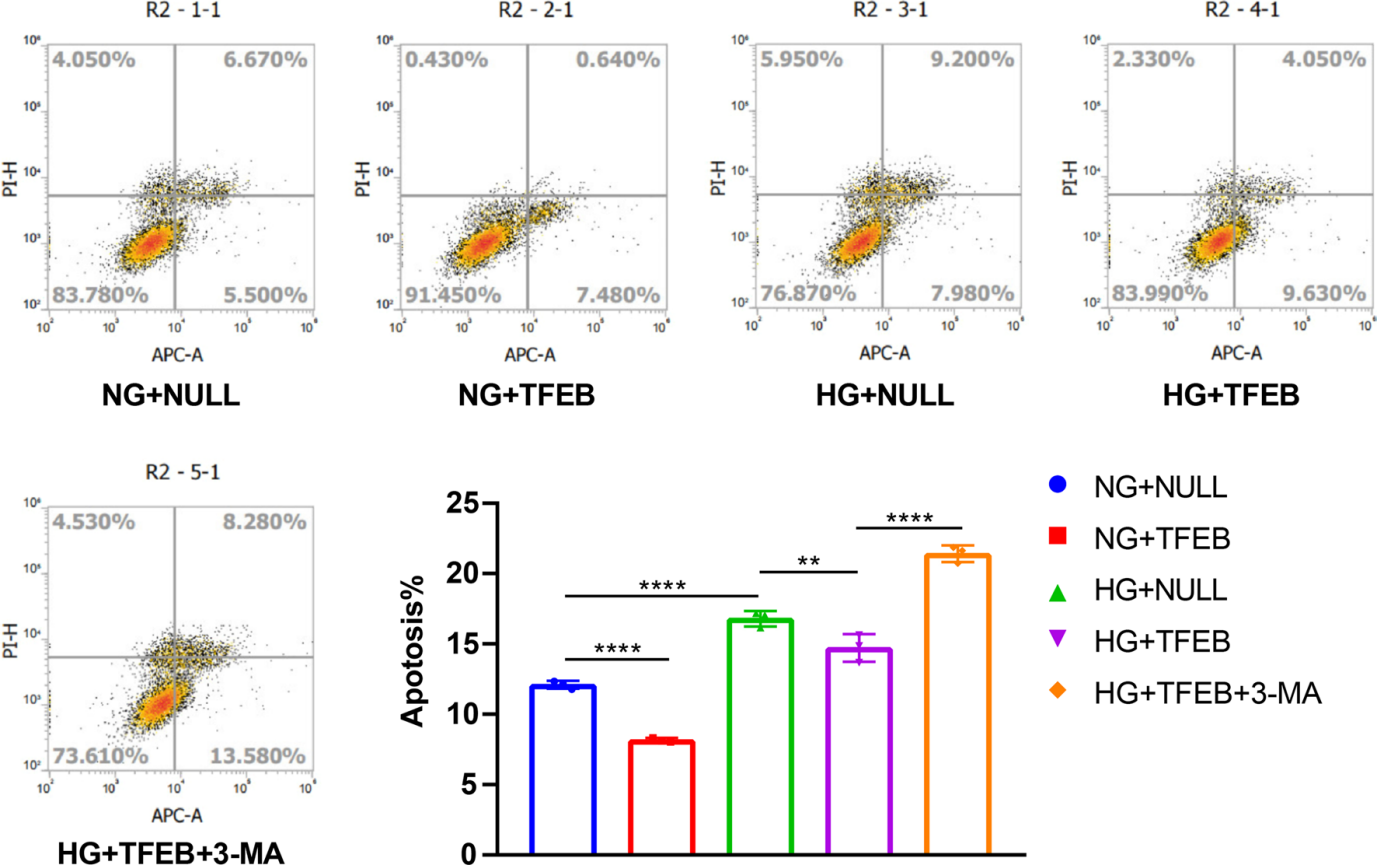
TFEB overexpression alleviated cell apoptosis of HG-cultured HT22 cells and the effect of TFEB was inhibited by 3-MA. HT22 cell apoptosis was assayed by flow cytometry, data are presented as the mean ± standard deviation (**A**), n = 3. *p < 0.05, **p < 0.01, ***p < 0.001, ****p < 0.0001

While there was no significant difference in cognition between the CON group and the CON group with overexpressed TFEB, T2DM mice with TFEB overexpression showed a significant improvement in cognition compared to those transduced with the empty vector (Fig. 4F–I). Furthermore, TFEB overexpression protected against cell apoptosis in NG or HG-cultured HT22 cells (Fig. 7A). These findings suggest that TFEB overexpression promotes the ALP-targeted clearance of AD-related proteins and alleviates DE.

### 3-MA treatment eliminated the positive effect of TFEB overexpression on DE

To verify that the therapeutic effect of TFEB overexpression is due to ALP activation, we conducted in vivo and in vitro experiments combining TFEB overexpression with 3-MA treatment. As expected, 3-MA inhibited ALP function, resulting in decreased ALP activity in T2DM mice (Fig. 4A, B) and HG-cultured HT22 cells (Fig. 5A, B) when used in combination with TFEB overexpression. Additionally, 3-MA blocked the enhanced autophagic flux effect of TFEB, as indicated by a decrease in the number of autophagosomes and autolysosomes (Fig. 5C–E). Furthermore, 3-MA treatment eliminated the positive effect of TFEB overexpression on AD-like pathology attenuation in vivo (Fig. 4C) and in vitro (Fig. 6A). This result was also confirmed by immunofluorescence staining analysis (Fig. 6B, C, F, G). Combined treatment of 3-MA and TFEB vector did not improve cognition in T2DM mice (Fig. 4F–I) or protect against cell apoptosis in HG-cultured HT22 cells (Fig. 7A), further supporting the idea that the therapeutic effect of TFEB overexpression on DE is mediated through ALP activation.

### TFEB knockdown inhibited ALP function and aggravated AD-like pathology

The results above demonstrate that increasing TFEB nuclear translocation, either through mTOR inhibition at the post-translational level or TFEB overexpression at the transcriptional level, can enhance ALP-targeted clearance of proteins related to AD. This underscores the therapeutic potential of TFEB in the context of neurodegenerative diseases. To further verify the pivotal role of TFEB in this process, gene interference was used to knock down TFEB.

**Fig. 8:**
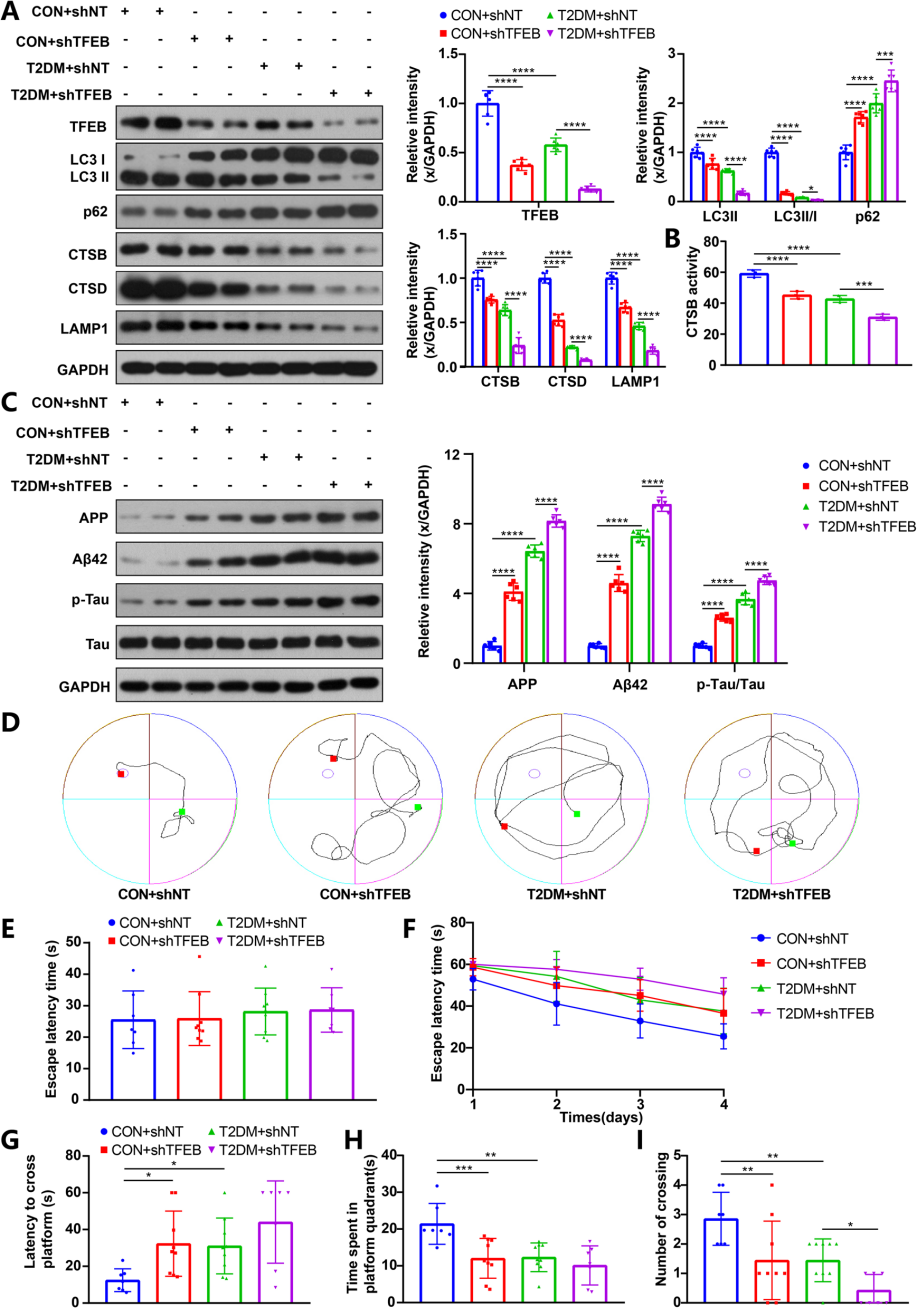
TFEB knockdown reduced ALP function, aggravated AD-like pathology and cognitive function of T2DM mice. TFEB, LC3, p62, CTSB, CTSD and LAMP1 were measured via western blotting in the hippocampus of mice (**A**), GAPDH was used as the loading control, n = 6. Results of CTSB activity of the mice, the activity of CTSB was expressed as fluorescent units/ug protein (**B**), n = 3. APP, Aβ42, p-Tau and Tau were measured via western blotting in the hippocampus of mice (**C**), GAPDH was used as the loading control, n = 6. Typical swimming traces of training days during the hidden platform test (**D**), the starting point was marked by the green square while the ending point was marked by the red square; the escape latency time of visual platform experiment, which showed that there was no obvious movement and visual disturbance in mice of each group (**E**); the escape latency time was analyzed during the hidden platform test (**F**); the latency of first arrival to targeted platform in probe trials (**G**), time spent in the targeted platform quadrant during the probe trials (**H**), the number of crossing targeted platform during the probe trials (**I**) was analyzed; n = 7–9. *p < 0.05, **p < 0.01, ***p < 0.001, ****p < 0.0001

**Fig. 9:**
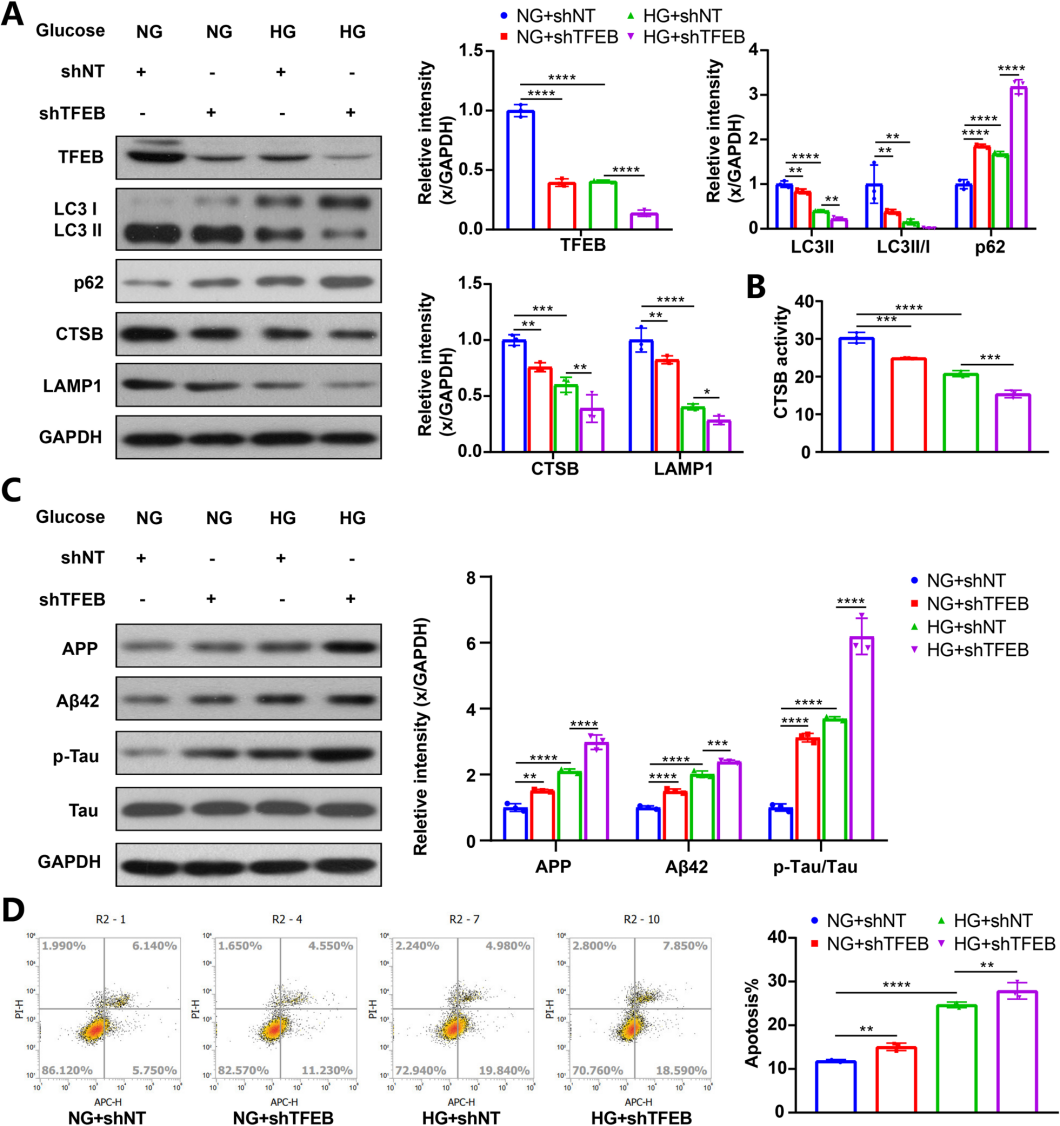
TFEB knockdown reduced ALP function, aggravated AD-like pathology and cell apoptosis of HG-cultured HT22 cells. TFEB, LC3, p62, CTSB and LAMP1 and were measured via western blotting in HT22 cells (**A**), GAPDH was used as the loading control, n = 3. Results of CTSB activity of the HT22 cells, the activity of CTSB was expressed as fluorescent units/ug protein (**B**), n = 3. APP, Aβ42, p-Tau and Tau were measured via western blotting in HT22 cells (**C**), GAPDH was used as the loading control, n = 3. HT22 cell apoptosis was assayed by flow cytometry, data are presented as the mean ± standard deviation (**D**), n = 3. *p < 0.05, **p < 0.01, ***p < 0.001, ****p < 0.0001

Western blot analysis confirmed the specific downregulation of TFEB expression in the hippocampus of mice and HT22 cells transduced with a shTFEB vector (Figs. 8A; 9A). TFEB downregulation significantly impaired ALP function, as evidenced by accumulated p62 proteins and decreased levels of LC3II proteins, LC3-II/I ratio, LAMP1, CTSB and CTSD proteins, as well as CTSB activity (Figs. 8A, B; 9A, B). Correspondingly, TFEB knockdown markedly aggravated AD-like pathology (Figs. 8C, 9C). Finally, TFEB knockdown deteriorated the cognition of mice (Fig. 8F–I) and also led to higher cell apoptosis (Fig. 9D).

These results suggest that TFEB knockdown can suppress ALP, exacerbate AD-like pathology, and ultimately worsen neurodegeneration, further highlighting the robust correlation between TFEB and ALP-targeted clearance of proteins related to AD.

## Discussion

DE is a well-accepted complication of T2DM, and the cognitive impairment caused by DE has drawn increasing attention from society. In this study, we used HFD/STZ-treated C57BL/6J mice as a T2DM model and demonstrated a significant decline in cognitive function. Additionally, we observed an increase in neuronal apoptosis, a direct cause of memory and learning impairment, in HT22 cells cultured with high glucose. Cognitive impairment in diabetic patients can significantly hamper their daily functioning and adversely affect their quality of life, making it imperative to explore the pathological mechanisms and effective therapeutic strategies for DE.

According to reports, the primary pathological mechanisms of DE include vascular factors and neurodegenerative changes, specifically AD-like pathology [51]. This study focuses on the latter. Excessive accumulation of Aβ can impair long-term potentiation, leading to synaptic dysfunction, neuronal loss, and subsequent memory deficits [52, 53]. Moreover, increased p-Tau can destabilize neuronal cytoskeletons and impair axonal transport, resulting in synaptic impairment and progressive neurodegeneration once a pathological threshold is reached [54]. When the balance between AD-related protein production and clearance is disrupted, proteins such as Aβ and p-Tau will deposit either extracellularly or intracellularly, causing massive neuronal loss. Our study confirms that overloaded AD-like pathology is present in both the hippocampus of T2DM mice and HG-cultured HT22 cells. Therefore, alleviating AD-like pathology is a promising therapeutic strategy for DE.

Previous studies have achieved significant success in treating DE by targeting AD-like pathology in various ways [13–15]. In this study, we focus on the role of ALP in the clearance of AD-related proteins. Recent studies have extensively examined the importance of ALP in AD, and they suggest that impaired ALP function leads to a decreased clearance of Aβ and p-Tau [20–26]. The ALP-targeted clearance mechanism involves the formation of autophagosomes or autophagic vacuoles, which isolate cytoplasmic contents. Subsequent lysosomal fusion facilitates the degradation of autophagosomes and their contents, including Aβ, APP, and p-Tau [55–58]. Impaired ALP function can lead to the accumulation of AD-related proteins, which can be toxic to brain cells, resulting in neuronal loss and cognitive impairment. Our study found that the function of ALP was significantly repressed in the hippocampus of T2DM mice and HG-cultured HT22 cells. After being treated with the autophagy inhibitor 3-MA, T2DM mice and HG-cultured HT22 cells exhibited even more severe AD-like pathology, accompanied by a decline in cognitive function and an increase in cell apoptosis levels. These findings support the association between impaired ALP function and aggravated AD-like pathology in DE. Therefore, the activation of ALP represents a promising approach for treating DE.

TFEB is a potent activator of ALP, and its nuclear translocation is closely associated with ALP function [28]. Emerging evidence suggests that TFEB may be a promising therapeutic target for some brain diseases, such as AD [59, 60] and ischemic stroke [29]. Our study found that TFEB nuclear translocation was markedly reduced in T2DM mice and HG-cultured HT22 cells, which is likely responsible for ALP impairment in the diabetic condition. Thus, increasing TFEB nuclear translocation appears to be a reasonable method for ALP activation.

Phosphorylation of TFEB, mainly mediated by mTOR, leads to its sequestration in the cytosol and is strictly associated with its nuclear translocation [28, 31]. In particular, we observed higher mTOR activity and p-TFEB levels in T2DM mice and HG-cultured HT22 cells. Therefore, the inhibition of mTOR activity is essential for increasing TFEB nuclear translocation, as it can reduce p-TFEB. Rapamycin is known for its ability to inhibit mTOR activity. Indeed, after rapamycin treatment, the mTOR activity and p-TFEB levels of T2DM mice and HG-cultured HT22 cells were reduced. Furthermore, rapamycin treatment improved ALP function and alleviated AD-like pathology, ultimately reducing cell apoptosis in HG-cultured HT22 cells and relieving cognitive impairment in T2DM mice. Overall, we suggest that, in the case of T2DM, the overactivation of mTOR leads to a reduction of TFEB in the nucleus. Thus, the activation of TFEB by inhibiting mTOR has a potential therapeutic effect on DE.

Previous studies have reported that transducing exogenous TFEB is an effective method to directly enhance TFEB content in the nucleus [29, 30]. Notably, we found that total TFEB levels decreased both in T2DM mice and HG-cultured HT22 cells. Thus, in addition to reducing TFEB phosphorylation at the post-translational level, we also regulated TFEB expression at the transcriptional level to increase TFEB content in the nucleus. As expected, ALP function was enhanced after TFEB overexpression. We further confirmed the vital role of TFEB overexpression on autophagy flux enhancement through mRFP-GFP-LC3 puncta analysis. Moreover, TFEB overexpression markedly alleviated AD-like pathology and increased the level of colocalization of LAMP1 with Aβ42 and LAMP1 with p-Tau in double immunofluorescence staining, indicating the importance of TFEB overexpression in the ALP-targeted clearance of AD-related proteins. In addition, TFEB overexpression lowered cell apoptosis and improved cognition in T2DM mice. However, the above effects were blocked when treated with 3-MA simultaneously, which further confirms that TFEB overexpression worked through ALP activation. These observations demonstrate that TFEB overexpression could serve as a therapeutic strategy for DE via ALP activation.

In further support of the central role of TFEB in DE, we knocked down TFEB both in vivo and in vitro. Obviously, TFEB knockdown suppressed ALP function, aggravated AD-like pathology, and ultimately elevated cell apoptosis levels and deteriorated cognition, confirming that TFEB plays an essential role in the development of DE.

This study demonstrates the feasibility of TFEB as a therapeutic target for DE. Based on in vivo and in vitro studies, we elucidate that increasing TFEB nuclear translocation, either through mTOR inhibition at the post-translational level or TFEB overexpression at the transcriptional level, is beneficial for DE treatment, due to its ability to enhance ALP-targeted clearance of AD-related proteins. This provides new insights for DE treatment. Meanwhile, considering the important role of TFEB in the development of DE, emerging drugs or chemicals targeted at increasing TFEB nuclear translocation, such as metformin [61] and trehalose [62], may have potential therapeutic effects on DE, which remain to be investigated in the future.

## Supplementary Information

Additional file 1: Figure S1. T2DM model established. Diagram illustrating experimental design. Results of IPGTT, plasma insulinand random blood glucoseof the mice, n = 7–10. *p < 0.05, **p < 0.01, ***p < 0.001, ****p < 0.0001.

Additional file 2: Figure S2. Immunofluorescence co-location staining of LAMP1 with Aβ42 and LAMP1 with p-Tau of mice. Representative double staining image of LAMP1 with Aβ42and LAMP1 with p-Tauwith or without TFEB overexpression, scale bar: 100 μm; the co-localization is represented by yellow signals and quantified as Pearson’s correlation coefficient, n = 5. *p < 0.05, **p < 0.01, ***p < 0.001, ****p < 0.0001.

## Data Availability

Data and materials are available from the corresponding author on reasonable request.

## Abbreviations

AAV: Adeno-associated virus
AD: Alzheimer's disease
ALP: Autophagy-lysosome pathway
APP: Amyloid precursor protein
Aβ: β-Amyloid peptides
CON: Control
CTSB: Cathepsin B
CTSD: Cathepsin D
DE: Diabetic encephalopathy
HFD: High fat diet
HG: High glucose
IACUC: Institutional Animal Care and Use Committee
LAMP1: Lysosomal-associated membrane protein 1
LC3: Microtubule-associated protein 1 light chain 3
mTOR: Mechanistic target of rapamycin
MWM: Morris Water Maze
NG: Normal glucose
PFA: Paraformaldehyde
p-Tau: Hyperphosphorylated tau
p62: Sequestosome 1
shTFEB: TFEB shRNA
SPF: Specific pathogen-free
STZ: Streptozocin
TFEB: Transcription factor EB
T2DM: Type 2 diabetes mellitus
3-MA: 3-Methyladenine
