# Supplementary figures and images for: mTOR-dependent TFEB activation and TFEB overexpression enhance autophagy-lysosome pathway and ameliorate Alzheimer's disease-like pathology in diabetic encephalopathy
Source: Cell Commun Signal. 2023 May 4;21:91. doi: 10.1186/s12964-023-01097-1 (PMC10158341; doi:10.1186/s12964-023-01097-1)

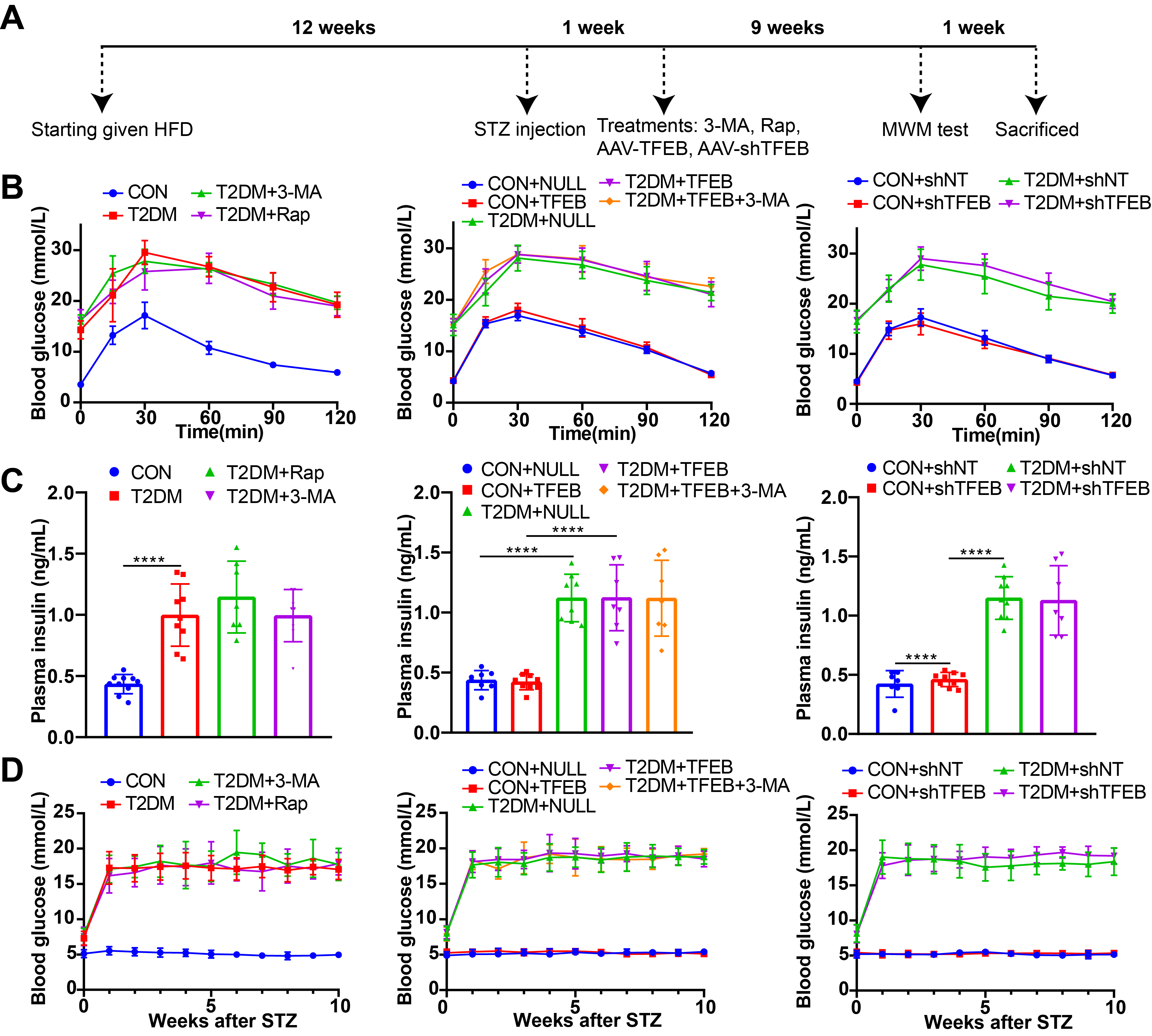

Supplement: Supplementary file 2 — Additional file 1: Figure S1. T2DM model established. Diagram illustrating experimental design. Results of IPGTT, plasma insulinand random blood glucoseof the mice, n = 7–10. *p < 0.05, **p < 0.01, ***p < 0.001, ****p < 0.0001. [file 12964_2023_1097_MOESM2_ESM.jpg]

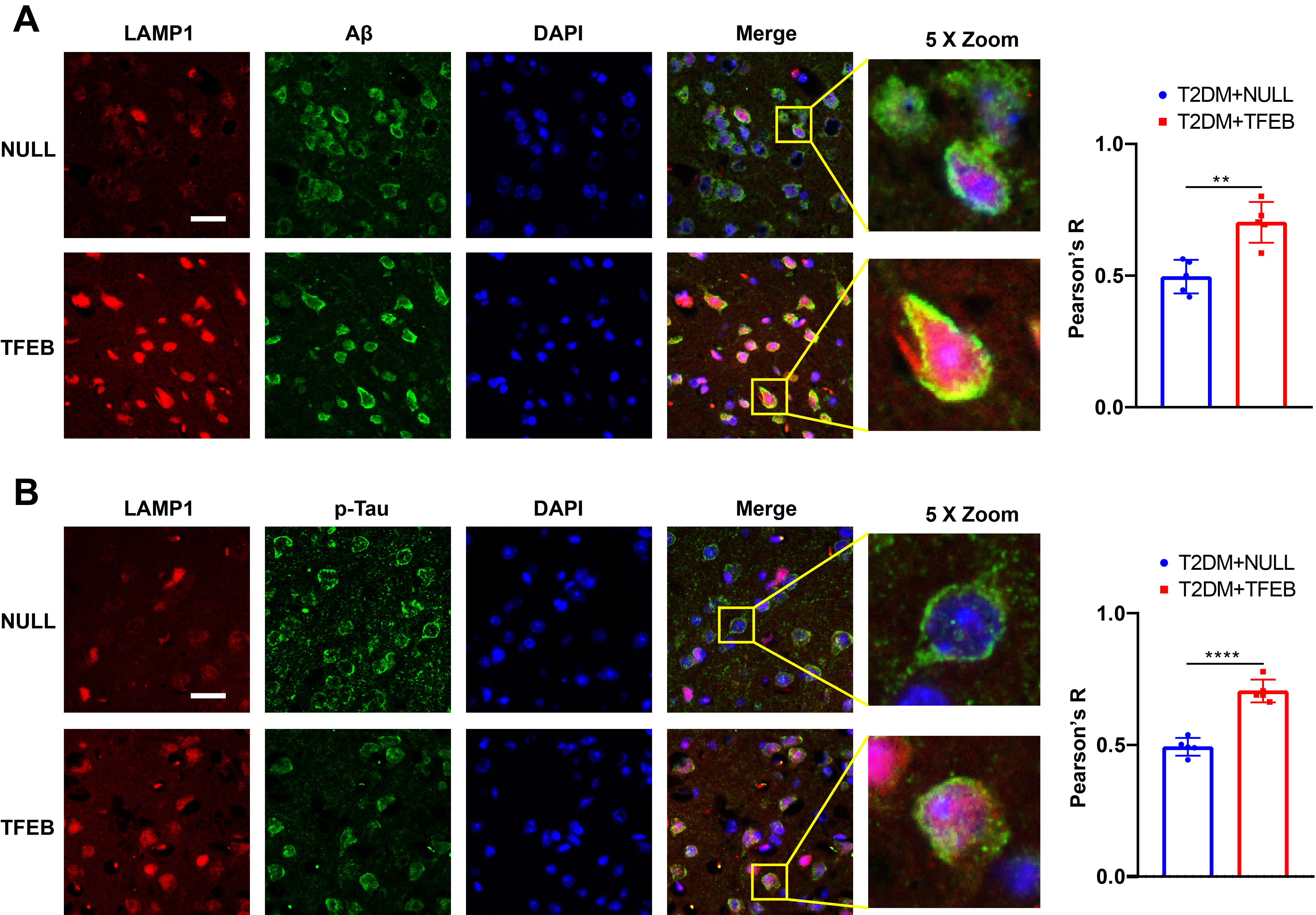

Supplement: Supplementary file 3 — Additional file 2: Figure S2. Immunofluorescence co-location staining of LAMP1 with Aβ42 and LAMP1 with p-Tau of mice. Representative double staining image of LAMP1 with Aβ42and LAMP1 with p-Tauwith or without TFEB overexpression, scale bar: 100 μm; the co-localization is represented by yellow signals and quantified as Pearson’s correlation coefficient, n = 5. *p < 0.05, **p < 0.01, ***p < 0.001, ****p < 0.0001. [file 12964_2023_1097_MOESM3_ESM.jpg]
